# W::Neo: A Novel Dual-Selection Marker for High Efficiency Gene Targeting in *Drosophila*

**DOI:** 10.1371/journal.pone.0031997

**Published:** 2012-02-13

**Authors:** Wenke Zhou, Juan Huang, Annie M. Watson, Yang Hong

**Affiliations:** Department of Cell Biology and Physiology, University of Pittsburgh School of Medicine, Pittsburgh, Pennsylvania, United States of America; University of Massachusetts Medical School, United States of America

## Abstract

We have recently developed a so-called genomic engineering approach that allows for directed, efficient and versatile modifications of *Drosophila* genome by combining the homologous recombination (HR)-based gene targeting with site-specific DNA integration. In genomic engineering and several similar approaches, a “founder” knock-out line must be generated first through HR-based gene targeting, which can still be a potentially time and resource intensive process. To significantly improve the efficiency and success rate of HR-based gene targeting in *Drosophila*, we have generated a new dual-selection marker termed W::Neo, which is a direct fusion between proteins of eye color marker White (W) and neomycin resistance (Neo). In HR-based gene targeting experiments, mutants carrying *W::Neo* as the selection marker can be enriched as much as fifty times by taking advantage of the antibiotic selection in *Drosophila* larvae. We have successfully carried out three independent gene targeting experiments using the W::Neo to generate genomic engineering founder knock-out lines in *Drosophila*.

## Introduction

We have recently developed a new approach termed genomic engineering that combines the gene targeting with phage integrase ϕC31-mediatd DNA integration for the purpose of directed, efficient and versatile modifications of endogenous genomic loci in *Drosophila* [1], [2]. Genomic engineering is a two-step process. First, a “founder” knock-out is generated by homologous recombination (**HR**)-based gene targeting that deletes the target locus and effectively replaces it with a ϕC31-attP integration site. Second, the target locus can then be modified into virtually any desirable knock-in alleles through ϕC31-mediated integration of corresponding DNA constructs into the founder line [1], [2]. We have also developed an additional integrase system for making sophisticated knock-in mutants by successive and targeted DNA integrations in genomic engineering [3]. Although generating novel knock-in alleles through site-specific DNA integration is extremely efficient compared to the HR-based knock-in/knock-out process, in genomic engineering and several similar approaches [1], [2], [4], [5], [6] a founder knock-out line must be first generated through HR-based targeting. In *Drosophila*, the frequency of HR for a given target locus can vary from ∼10^−2^ to ∼10^−6^, *i.e.* an approximately 10,000-fold difference [2], [7]. For target loci that are of <10^−4^ HR frequency, targeting experiments can be highly time and labor intensive. Therefore, more efficient and reliable gene targeting remains crucial for approaches like genomic engineering.

**Figure 1:**
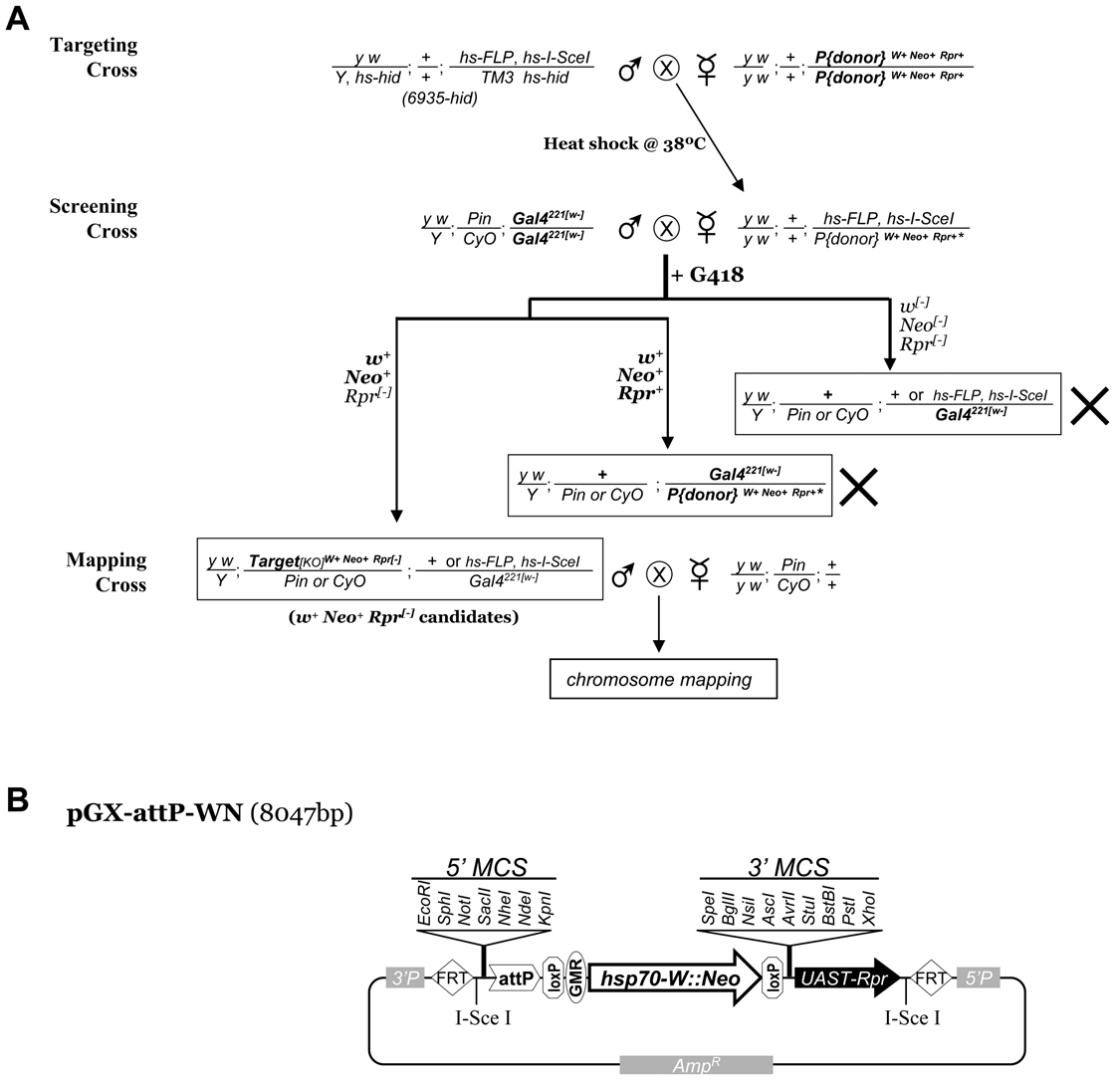
Application of multiple selections in gene targeting. **A**. Genetic crosses of ends-out targeting based on the dual positive screening of *w+* and *Neo+* for targeting candidates, together with the negative selection of UAS-Rpr (Rpr+) for eliminating false positives. “X”: genotypes eliminated or greatly reduced in numbers by G418 selection or UAS-Rpr counter-selection. **B**. Map of pGX-attP-WN. pGX-attP-WN is a P-element based transforming vector. *5′P* and *3′P*: 5′ and 3′ P-element sequences; Amp^R^: ampicillin-resistant gene.

General HR-based gene targeting in *Drosophila* [8], [9] requires several rounds of genetic crosses including **targeting cross**, **screening cross**, and **mapping cross** (Figure 1A) [7]. Transgenic flies of targeting construct were first generated to carry the donor DNA as a chromosomal insertion (“P[donor]”) flanked by FRT sites. To initiate the homologous recombination, the donor DNA in *P[donor]/hs-FLP hs-I-SceI* of targeting cross progeny is excised and linearized by heatshock-induced expression of Flipase (FLP) and the restriction enzyme I-SceI. In screening cross, heatshocked *P[donor]/hs-FLP hs-I-SceI* targeting females are crossed with *w^[−]^* males so potential targeting mutant progeny may be recovered based on the dominant *w+* marker (i.e. red eye). Mapping cross will be used to further genetically map and confirm the targeting mutants. In order to improve the scalability and throughput in these major genetic crosses, we have in the past developed several measures such as optimizing targeting vectors and *hs-FLP hs-I-SceI* stocks and introducing a *UAS-Rpr* negative selection marker (Figure 1A) [7]. These improvements have already yielded a high success rate for a number of targeting experiments [2], [7]. Nonetheless, for targeting experiments of <10^−4^ HR frequency, >10^5^ progeny from screening cross have to be screened visually based on eye color marker *w+*. This time and labor intensive process directly limits the scale of targeting experiments.

To solve this problem, increasing the HR frequency by the means such as target-specific zinc finger nuclease (ZFN) likely presents one of the most promising approaches [10]. Nonetheless at present target-specific ZFNs or similar nucleases require extensive testing and refining efforts that to large degree may offset the benefits of increased HR frequency [11]. As an alternative, more efficient screening methods can also significantly increase the success rate of gene targeting. To this end, we developed an approach to directly enrich the targeting mutants by introducing a dominant selection marker *Neo* [12] in addition to the *w+*.

## Results and Discussion

We took advantage of the well established fact that *Drosophila* larvae are highly sensitive to G418 which is a drug related to neomycin and karamycin, but can be made resistant by the expression of neomycin resistance gene (Neo) [12]. By making candidate mutants neomycin-resistant (*Neo+*), G418 can be used to directly eliminate the vast number of screening cross progeny carrying no targeting events (Figure 1A). This approach provides several advantages. First, G418 can be easily added to the fly food, thus is fully compatible with the current genetic cross schemes of gene targeting. Second, G418-sensitivity in *Drosophila* larvae is dosage dependent [12]. By administrating G418 at pre-determined concentrations it is possible to eliminate a large percentage (*e.g.* 90%–99%) but not all of the larvae, minimizing the risk of killing *Neo+* targeting mutants while at the same time leaving enough number of survival larvae to ensure healthy growing conditions.

**Figure 2:**
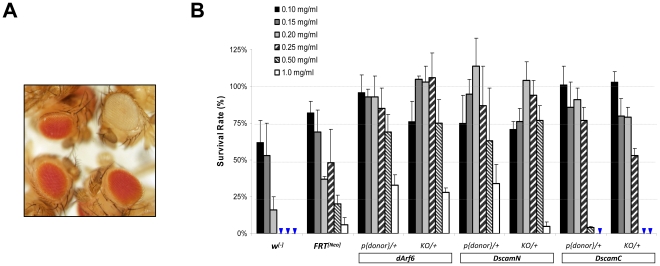
Expression of *W::Neo* confers both *w+* and G418-resistance in flies. **A**. Eye color in representative males from (clockwise from top right): *w^1118^*, *y w; pArf6^GX22^/TM3*, *y w; pDscam-N^GX113^/TM3*, and *y w; pDscam-C^GX1^/TM3*. **B**. G418-resistance of *W::Neo* transgenic donor lines and founder knock-out flies. Arrowheads indicate 0% survival rate. *FRT^[Neo]^*: *y w; FRT-42D ubi::GFP^NLS^/+*; *P{donor}*: transgenic donor insertion; *KO*: targeting allele; *w^[−]^*: *TM3/+* and *CyO/+* cross progeny. See Materials and Methods for detail genotypes.

Although *Neo+* would be an effective marker for enriching targeting mutants, *w+* is still the most convenient marker for genetic mapping. To incorporate both *w+* and *Neo+* into the targeting mutants, we made a *white::Neo* (*W::Neo*) gene encoding a chimeric protein in which Neo is directly fused to the C-terminus of W+. This design also minimized the size of the *w+ Neo+* dual selection marker for potentially more efficient molecular cloning, donor DNA excision and HR. To test the effectiveness of W::Neo for being both *w+* and *Neo+*, we first generated a pKIKO-WN vector by replacing the *w+* in an older targeting vector pKIKO [7] with *W::Neo*. Through standard P-element based transgenesis, we obtained several *w+* transgenic lines of pKIKO-WN showing that *W::Neo* functioned as a normal *w+* marker for producing red eye flies (see Figure 2A). We also picked one of the pKIKO-WN lines and confirmed that its *w+* progeny showed clear resistance to neomycin compared to their *w^[−]^* siblings carrying no pKIKO-WN (Table S1).

To test the effectiveness of *W:Neo* marker in targeting experiments, we carried out three ends-out (replacement) gene targeting experiments using W::Neo as a dual selection marker (Table 1). We first modified the pRK2 based gene targeting constructs of *dArf6* [7] by replacing the *w+* with *W::Neo*. We obtained 16 *w+* transgenic donor lines by injecting 1150 embryos. As shown in Table S2, all but three lines showed clear resistance to G418 at 0.20 mg/ml concentration. The three lines that showed reduced resistance to G418 also showed failures in FRT or Cre-mediated excisions and dramatically reduced effectiveness of UAS-Rpr negative selection marker, suggesting these components were likely damaged during transgenic insertion. We picked line#22 (*pArf6GX22*) to carry out the targeting experiment. Based on the experiments in Figure 2B, we determined that 0.20 mg/ml should be the optimal G418 concentration that eliminates >90% of *w^[−]^* sibling larvae with apparently no effect on the survival of *pArf6GX22/+* larvae.

**Table 1:** Design of gene targeting for *dArf6*, *Dscam-N* and *Dscam-C* founder Knock-out lines.

Target Gene	Target Chromo-some	Exons/mRNA Isoforms	5′+3′ Arms* (kb)	Targeted gDNA Deletion**	Genomic Deletion Size (kb)	Protein Deletion/Full Length (aa)
*dArf6*	2^nd^	3/5	4.5+3.1	*2R: 11,210,875–11,213,032*	2.157	175/175
*Dscam-N*	2^nd^	24/38016	5.5+3.2	*2R:3249024—3254750*	5.727	108/2037
*Dscam-C*	2^nd^	24/38016	5.3+3.2	*2R:3206840—3214484*	7.645	439/2037

*: 5′+3′ Arms: the lengths of 5′ and 3′ homology arms in targeting construct.

**: According to *Drosophila* genome release FB2011.07 at www.flybase.org.

Using our improved targeting stocks [7], we were able to collect 20,000 targeting females of *pArf6GX22/hs-FLP hs-I-SceI* from targeting crosses (See Figure 1A). To set up the screening cross, 12,000 targeting females were crossed with *w; Gal4^4-77[w−]^* [7]. The Gal4 drives the expression of *UAS-Rpr* negative selection marker that eliminates >96% of false positives [7]. Half of the cross population were grown on normal food without G418. As previously reported we screened ∼700,000 progeny and recovered five targeting mutants [7] (Table 2). Another half of the cross population containing 6,000 targeting females were grown on food containing 0.20 mg/ml G418. Based on the tests conducted in Table S3, we set up the crosses in G418 bottles with 30 females per bottle. As expected, G418 drastically reduced the number of progeny produced. In total we collected and screened only ∼67,000 flies but recovered 23 targeting mutants (Table 2). Extrapolating from such data, using W::Neo marker with G418 selection we enriched the targeting mutant frequency from 5/(7×10^5^) to 23/(6.7×10^4^), an enrichment of approximately 48 times. The mere 67,000 flies we screened were effectively equivalent to >3×10^6^ screening cross progeny without G418 selection.

**Table 2:** Generation of founder knock-out lines by ends-out targeting.

Target Gene	G418 (mg/ml)	Targeting Virgins Females	Screening Cross Progeny^a^	Preliminary Candidates	On Target Chr.	Genetically Verified	PCR Verified	HR Frequency^b^
***dArf6***	0	6,000	∼7×10^5^	315	30/315	5/30^d^	5/5	∼7×10^−6^
	0.20	6,000	∼6.7×10^4^	221	43/221	23/43^d^	6/6	*∼3.4×10^−4^*
***Dscam-N***	0	16,000^c^	∼1.6×10^5^	71	50/71	23/50^e^	2/2	∼1.4×10^−4^
	0.20	(16,000)^c^	∼3.3×10^4^	557	399/557	162/399^e^	5/5	*∼4.9×10^−3^*
***Dscam-C***	0	9,400^c^	∼1.9×10^5^	23	11/23	3/11^e^	3/3	∼1.6×10^−5^
	0.20	(9,400)^c^	∼2.2×10^4^	42	12/42	3/12^e^	3/3	*∼1.3×10^−4^*

**^a.^**Total estimated number of screening cross progeny screened in each targeting experiment. Because progeny of multiple vials or bottles were pooled and screened together, we did not register the clonality of the preliminary candidates. We assumed that each targeting mutant obtained was due to a distinct targeting event, based on the low HR frequency observed.

**^b.^**Since all female candidates were discarded in targeting experiments, the adjusted HR frequency should be twice higher than listed here.

**^c.^**Screening crosses were set up on the normal food first, then transferred to G418 food after two days.

**^d.^**A *dArf6^ΔKG#1^* deletion allele generated by P-excision was used for complementation assays [7].

**^e.^**Null allele of *P{PZ}Dscam^05518^* (BL#11412) [13] was used for complementation assays.

**Figure 3:**
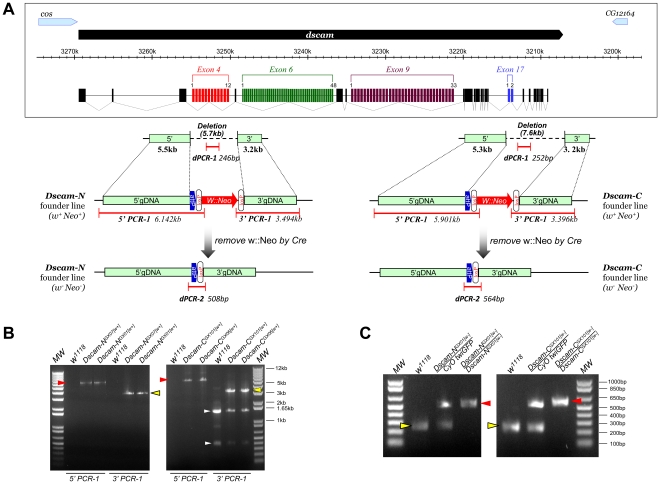
Gene targeting of *Dscam-N* and *Dscam-C*. **A**. Targeting design and PCR verification of *Dscam-N* and *Dscam-C* founder lines. Boxed are the genomic DNA (gDNA) structure and alternative-splicing patterns of *Dscam* locus. *Dscam* locus contains four alternative-splicing exons: 4, 6, 9 and 17 [13]. Green boxes are gDNA regions used for 5′ and 3′ homology arms in the targeting constructs. In the *Dscam-N* founder knock-out line, a 5.7 kb genomic DNA covering the alternatively spliced exon 4 are deleted. In the *Dscam-C* founder knock-out line, a 7.6 kb genomic DNA covering the alternatively spliced exon 17 plus all the remaining downstream exons and 3′UTR are deleted. *Dscam-N* and *Dscam-C* founder knock-out lines carrying *W::Neo* marker are verified by 5′ and 3′ PCRs. 5′ or 3′ PCR is designed with one primer annealing within the W::Neo, while another primer anneals outside the gDNA region used for homology arms (“5′ gDNA” or “3′ gDNA”) in targeting constructs. Thus, only the correct targeting events will yield PCR products of expected size. *Dscam-N* and *Dscam-C* founder lines with *W::Neo* removed are further verified by dPCR-1 and dPCR-2. dPCR-1 is located within, while dPCR-2 spans over, the deleted region of *Dscam-N* or *Dscam-C*. **B**. 5′ and 3′ PCR-1 (red and yellow arrowheads, respectively) results from adults of *Dscam-N^GX07[w+]^/CyO, Dscam-N^GX01[w+]^/CyO, Dscam-C^GX101[w+]^/CyO* and *Dscam-C^GX06[w+]^/CyO*. *w^1118^* was used as wild type control. White arrowheads pointing to non-specific PCR products. **C**. dPCR-1 (yellow arrowhead) and dPCR-2 (red arrowhead) results from embryos of *Dscam-N^GX01[w−]^* and *Dscam-C^GX101[w−]^* with *W::Neo* removed. *Dscam-N^GX01[w−]^* and *Dscam-C^GX101[w−]^* were balanced on *CyO, twi-GAL4, UAS-2xEGFP* (“CyO twiGFP”) chromosome so homozygous embryos could be distinguished by the absence of GFP. *w^1118^* was used as the wild type control. For each PCR reaction genomic DNA was prepared by pooling approximately ten embryos together. For each sample, dPCR-1 and dPCR-2 reactions were carried out separately and were pooled before loading on the gel. MW: 1kb-plus DNA ladder (from Invitrogen); 5′ and 3′: the 5′ and 3′ homology arms of *Dscam-N* and *Dscam-C* targeting construct.

We then carried out two new targeting experiments against the *Dscam* locus using the pGX-attP-WN targeting vector (Figure 1B). *Dscam* encodes a neuronal adhesion molecule of extraordinary diversity through alternative splicing (Figure 3A) [13]. Based on the genomic engineering approach, we targeted the deletions of exon#4 and #17 to generate two different founder knock-out lines designated as *Dscam-N* and *Dscam-C*, respectively. All ten *Dscam-N* and three *Dscam-C* transgenic donor lines were resistant to at least 0.25 mg/ml G418 (data not shown). For the donor lines used for carrying out the targeting experiments, *pDscam-NGX113* showed G418-resistance similar to *pArf6GX22* whereas *pDscam-CGX1* appeared to be sensitive to ∼0.50 mg/ml G418 (Figure 2B).

For the *Dscam-N* targeting, we set up a screening cross using 16,000 targeting females in 800 vials with normal food (i.e. 20 females per vial). After three days we transferred flies to bottles of ∼0.20 mg/ml G418 food, at the density of 160 females per bottle. The flies were transferred to new G418 bottles every three to four days. The normal food culture yielded ∼1.6×10^5^ progeny and we recovered 23 targeting candidates (Table 2). In contrast, G418 bottles yielded 3.3×10^4^ progeny but we recovered 162 targeting mutants. The *Dscam-C* targeting was carried out similarly. We recovered 3 targeting mutants from 1.9×10^5^ progeny yielded from normal food, and 3 from ∼2.2×10^4^ progeny yielded from G418 food (Table 2). Based on screening the progeny from normal food, the HR frequency for *Dscam-N* targeting can be estimated is ∼1.4×10^−4^, while for *Dscam-C* is ∼1.6×10^−5^. Overall, the enrichment of targeting mutants by G418 selection can be roughly estimated as 34 and 9 times in our *Dscam-N* and *Dscam-C* targeting experiments, respectively. These numbers are likely underestimated due to the fact we grew G418 bottles under extremely overcrowded conditions of 160 females per bottle due to constrained incubator space at the time of experiments. As expected, both *Dscam-N* and *Dscam-C* mutants are lethal and their lethality can be rescued by integrating back the deleted fragment of gDNA into their corresponding knock-out founder lines (data not shown).

It should be noted that the *hsp70* promoter which drives the W::Neo expression in targeting mutants is not transcriptionally insulated and although its expression in eyes are boosted with an eye-specific GMR enhancer [7] (Figure 1B) its expression levels in other larval tissues still could suffer from chromosomal location-effects. Therefore, one potential caveat with G418 selection is that it may be difficult to know the actual strength of G418 resistance of a given targeting mutant. To investigate this issue, we systematically and quantitatively measured the G418 resistance of *dArf6*, *Dscam-N* and *Dscam-C* transgenic donor lines and targeting mutants. As shown in Figure 2B, all the *W::Neo* lines showed resistance well above 0.20 mg/ml G418, better than the common FRT lines carrying *hs-Neo* [14]. Assuming the results from the three target loci are representative, the risk of killing the real mutants should be very low at the 0.20 mg/ml G418 concentration that we used. In addition, for a given target locus there appears to be a good correlation between the transgenic donor line and the targeting mutants in terms of their G418-resistances (Figure 2B), consistent with the fact that in both lines the *W::Neo* is flanked by the same 5′ and 3′ gDNA which likely influence the expression of *W::Neo* the most. Therefore, by carefully testing the G418-resistenace in transgenic donor lines, it is possible to estimate the strength of G418-resistance of the future targeting mutants in order to optimize the G418 concentration for each individual targeting experiment. In general, 0.20 mg/ml of G418 seems to be working well in our experiments.

One practice we would recommend is to set up the screening crosses on normal food first, and transferring them to G418 food after one or two days. This method apparently gives healthier crosses on G418 food. In addition, the progeny from normal food come out earlier and can be used for a small to medium scale (∼10^4^–10^5^ flies) screening first. If no or not enough number of candidates are recovered, the target locus might be of low HR frequency and one may continue to screen the G418-selected progeny. In addition, despite that the *hsp70* promoter used in pGX-attP-WN is constitutively expressed, its expression level can still be greatly increased (up to one hundred-fold) by heat-shock [12]. Although we did not carry out heat-shock treatments in screening crosses, it can be easily adapted into the protocol. Finally, for targeting loci that may severely represses *hsp70* promoter, we are considering making modified targeting constructs that may feature stronger or insulated promoters.

In summary, we report here the successful applications of a novel *w+*/Neo+ dual selection marker that may effectively enrich the targeting mutants up to fifty times with the help of G418-selection. Our new pGX-attP-WN targeting vector could significantly facilitate the large scale targeting experiments, making target loci of <10^−6^ HR frequency much more experimentally accessible. Besides gene targeting, the W::Neo marker should be useful in routine *Drosophila* genetic crosses when both *w+* and *Neo+* are desirable for selecting a particular genotype.

## Materials and Methods

### Fly stocks and genetics

*y w/Y, hs-hid; hs-FLP, hs-I-SceI/TM3 e Sb hs-hid* (“6935-hid” BL#25679) and *y w; Pin/CyO; Gal42-21[w−]* (BL#26259) were generated previously [7]:

Following stocks were obtained from the Bloomington stock center: *y^1^ w^67c23^ P{1b; noc^Sco^/CyO* (BL#766); y^1^ w^67c23^ P{Crey}1b; D*/TM3, Sb (BL#851); *w^1118^; P{70FLP}10 (BL#6938); P{PZ}Dscam^05518^ cn^1^/CyO; ry^506^* (BL#11412); *y w; FRT-42D ubi-GFP^NLS^/CyO* (BL#5626); *w^1118^; In(2LR)Gla, wg^Gla-1^/CyO, P{GAL4-twi.G}2.2, P{UAS-2xEGFP}AH2.2* (BL#6662).

### DNA Constructs

The W::Neo marker was made by fusing the Neo coding sequence to the C-terminus of W+ in pKIKO vector [7] through overlapping PCR. Cloned W::Neo fragments were sequenced to ensure error-free PCR. pGX-attP-WN was made by replacing the coding sequence of *w+* in pGX-attP with W::Neo. Targeting construct of *dArf6* was described previously [7]. Molecular cloning of targeting constructs of *Dscam-N* and *Dscam-C* was carried out according to the protocols described in *Huang et al* [7]. Primers used for making targeting constructs are listed in Table S4. We used “cis-analyst” tool at http://www.fruitfly.org/seq_tools/other.html to compare genomic sequences between *Drosophila melanoganster* and *Drosophila pseudoobscura* to identify apparently non-conserved non-coding regions for positioning the ϕC31-attP and loxP sites in the target locus.

### Transgenesis and ends-out targeting

All transgenic flies were created using *w^1118^* stocks via the standard *P*-elements-based transgenic protocol. Most fly cultures and crosses were carried out at room temperature (∼22°C) or 25°C. Ends-out gene targeting and PCR-verifications of targeting candidates were carried out as described in *Huang et al* [7]. Primers used for PCR verifications as shown in Figure 3B,C are listed in Table S4.

### G418 treatment and tests

G418 (from Fisher Scientific) was directly added to microwave-melted fly food at ∼50°C as described [12]. All G418 concentrations reported here were effective concentrations based on the manufacture specifications. To quantitatively measure the G418 resistance, males from the following stocks were crossed with virgin females of: *w^1118^* (wild type control); *y w; pArf6^GX22^/TM3* (transgenic donor line used for *dArf6* targeting); *y w; pDscam-N^GX113^/TM3* (transgenic donor line used for *Dscam-N* targeting); *y w; pDscam-C^GX1^/TM3* (transgenic donor line used for *Dscam-C* targeting); *y w; dArf6^GX16[w+]^/CyO* (*dArf6* founder knock-out line); *y w; Dscam-N^GX07[w+]^/CyO* (*Dscam-N* founder knock-out line); *y w; Dscam-C^GX101[w+]^/CyO* (*Dscam-C* founder knock-out line); *y w; FRT-42D ubi-GFP^NLS^/CyO*;

For each cross, embryos were collected under 18°C for 24 hours and were split evenly into two vials containinig normal food (*i.e.* 0 mg/ml G418) and food of specified G418 concentration, respectively. On average approximately 200 embryos were placed in each vial. Adult *w+* and *w^[−]^* progeny were counted from each vial within 18 days under 25°C. For either *w^[−]^* or *w+* progeny, their survival rate in G418 selection is calculated as the percentage of (# from G418 food)/(# from normal food). Each test was carried out in at least triplicates. To calculate the *w^[−]^* survival rates in Figure 2B, we averaged the survival rates of all *TM3/+* and *CyO/+* cross progeny at a given G418 concentration.

## Supporting Information

Table S1 **W::Neo marker confers G418 resistance in transgenic flies.** * In each vial, six males of *y w/Y; pKIKO-WN#2/CyO* were crossed with six *y w; Pin/CyO* virgin females and were grown for five days. Progeny were scored based on *w+* marker. In the absence of G418 selection one third of the progeny were expected to be *w^[−]^* (i.e. *w^[−]^*/*w+* = 50%). ** These experiments were repeated three times. In experiment #2 only two *y w; pKIKO-WN#2/CyO* females were used for each cross, hence the small number of progeny. ***Avg***: Average.
(DOC)

Table S2 **G418-resistance test of** ***dArf6*** **transgenic donor lines.** ***a***: Tests were carried out similarly as in Table S1. ***b, c*** *: hs-FLP* and *hs-Cre* stocks used here constitutively express FLPase or Cre recombinase. In *hs-FLP* and *hs-Cre* crosses, constitutively expressed FLPase or Cre will excise the donor DNA, resulting in visible eye color variegation in the cross progeny. The degree of eye color variegation due to the loss of *w+* was compared by estimating the percentage of white area in each eye. 0% white area suggests likely at least one of the two FRT or loxP sites were damaged in the transgenic donor DNA insertion. ***d*** *: Gal4^2–21^* drives UAS-Rpr expression of the transgenic donor DNA that results in 100% lethality in the cross progeny. Significantly reduced lethality suggests either damaged transgenic donor DNA insertion or severe repression of UAS-Rpr expression due to chromosomal location. ***e*** *:* These three lines likely have damaged transgenic donor DNA insertion based on their test results with *hs-FLP, hs-Cre* and *Gal4^2–21^*. They also show much higher percentage of *w^[−]^* progeny on 0.25 mg/ml G418, presumably due to the fact that reduced G418-resistance in the *w+* progeny allowed better survival of *w^[−]^* siblings. ***f*** *:* The transgenic donor line used for targeting. ***n.d.***: Not done **Chr.**: chromosomal location of the transgenic insertion.
(DOC)

Table S3 **Optimal culture density for G418 selection in** ***dArf6*** **targeting.** Crosses were first set up in vials, and were then transferred to G418 bottles after one or two days. It appears that between 20 to 40 females per bottle, the yield of *w+* candidates per 100 targeting females remains relatively stable. We decided that crosses of 30 targeting females per bottle appears to be a good compromise between achieving the maximum recovery of *w+* candidates and minimizing the number of G418 bottles.
(DOC)

Table S4 **Primers used for the generation and verification of** ***Dscam-N*** **and** ***Dscam-C*** **founder knock-lines.** *: Primers for PCR verifications of *dArf6* founder lines were in *Huang et al* [7]. ***w+***: PCR for verifying founder knock-out lines that contain the *w+* marker. ***w^[−]^***: PCR for verifying founder lines that had their *w+* marker removed by loxP recombination. n/a: not applicable.
(DOC)
