# Supplementary material for: W::Neo: A Novel Dual-Selection Marker for High Efficiency Gene Targeting in Drosophila
Source: PLoS One. 2012 Feb 13;7(2):e31997. doi: 10.1371/journal.pone.0031997 (PMC3278458; doi:10.1371/journal.pone.0031997)
Supplement: Table S1 — W::Neo marker confers G418 resistance in transgenic flies. * In each vial, six males of y w/Y; pKIKO-WN#2/CyO were crossed with six y w; Pin/CyO virgin females and were grown for five days. Progeny were scored based on w+ marker. In the absence of G418 selection one third of the progeny were expected to be w[−] (i.e. w[−]/w+ = 50%). ** These experiments were repeated three times. In experiment #2 only two y w; pKIKO-WN#2/CyO females were used for each cross, hence the small number of progeny. Avg: Average. (DOC) [file pone.0031997.s001.doc]

**Table S1.**  W::Neo marker confers G418 resistance in transgenic flies.

| **G418**  (mg/ml) | | **0** | **0.15** | **0.375** |
| --- | --- | --- | --- | --- |
| ***%***  ***(w^[-]^/w+)*** *** | *#1*** | 46.4% (83/179) | 22.2% (47/212) | 1.6% (3/184) |
|  | *#2*** | 48.0% (12/25) | 10.7% (9/84) | 0.0% (0/37) |
|  | *#3*** | 68.3% (112/164) | 28.4% (44/155) | 2.7% (5/184) |
|  | ***Avg*** | **54.2%** (+/-12.2%) | **20.4%** (+/-9.0%) | **1.5%** (+/-1.5%) |

* In each vial, six males of *y w/Y; pKIKO-WN#2/CyO* were crossed with six *y w; Pin/CyO* virgin females and were grown for five days. Progeny were scored based on *w+* marker. In the absence of G418 selection one third of the progeny were expected to be *w^[-]^* (i.e. *w^[-]^*/*w+* = 50%)

** These experiments were repeated three times. In experiment #2 only two *y w; pKIKO-WN#2/CyO* females were used for each cross, hence the small number of progeny.

***Avg***: Average
