# Supplementary material for: W::Neo: A Novel Dual-Selection Marker for High Efficiency Gene Targeting in Drosophila
Source: PLoS One. 2012 Feb 13;7(2):e31997. doi: 10.1371/journal.pone.0031997 (PMC3278458; doi:10.1371/journal.pone.0031997)
Supplement: Table S2 — G418-resistance test of dArf6 transgenic donor lines. a: Tests were carried out similarly as in Table S1. b, c : hs-FLP and hs-Cre stocks used here constitutively express FLPase or Cre recombinase. In hs-FLP and hs-Cre crosses, constitutively expressed FLPase or Cre will excise the donor DNA, resulting in visible eye color variegation in the cross progeny. The degree of eye color variegation due to the loss of w+ was compared by estimating the percentage of white area in each eye. 0% white area suggests likely at least one of the two FRT or loxP sites were damaged in the transgenic donor DNA insertion. d : Gal42–21 drives UAS-Rpr expression of the transgenic donor DNA that results in 100% lethality in the cross progeny. Significantly reduced lethality suggests either damaged transgenic donor DNA insertion or severe repression of UAS-Rpr expression due to chromosomal location. e : These three lines likely have damaged transgenic donor DNA insertion based on their test results with hs-FLP, hs-Cre and Gal42–21. They also show much higher percentage of w[−] progeny on 0.25 mg/ml G418, presumably due to the fact that reduced G418-resistance in the w+ progeny allowed better survival of w[−] siblings. f : The transgenic donor line used for targeting. n.d.: Not done Chr.: chromosomal location of the transgenic insertion. (DOC) [file pone.0031997.s002.doc]

**Table S2. G418-resistance test of *dArf6* transgenic donor lines.**

| **Donor Lines** | **Chr.** | **G418** (~0.25mg/ml)***^a^*** | **x hs-FLP**  (eye color variegation) ***^b^*** | **x hs-Cre**  (eye color variegation) ***^c^*** | **x Gal4^2-21^**  (% lethality) ***^d^*** |
| --- | --- | --- | --- | --- | --- |
| pArf6^GX1^ | *n.d.* | + | 10% | 100% | 100% |
| pArf6^GX2^ | *n.d.* | + | *n.d.* | 100% | 100% |
| ***^e^*** pArf6^GX3^ | *n.d.* | (-) | 0% | 0% | 60% |
| pArf6^GX4^ | 3rd | + | 10% | 100% | 100% |
| ***^e^*** pArf6^GX5^ | *n.d.* | (-) | 0% | 0% | 50% |
| pArf6^GX6^ | *n.d.* | + | 5% | 100% | 100% |
| pArf6^GX7^ | *n.d.* | + | 30% | 100% | 100% |
| ***^e^*** pArf6^GX8^ | 3rd | (-) | 10% | 100% | 10% |
| pArf6^GX9^ | 2nd | + | 50% | 100% | 100% |
| pArf6^GX10^ | 3rd | + | 10% | 100% | 100% |
| pArf6^GX11^ | *n.d.* | + | 20% | 100% | 100% |
| pArf6^GX21^ | X | + | 10% | 100% | 100% |
| ***^f^* pArf6^GX22^** | **3rd** | + | **15%** | **100%** | 100% |
| pArf6^GX23^ | *n.d.* | + | 20% | 100% | 100% |
| pArf6^GX24^ | *n.d.* | + | 10% | 100% | 100% |
| pArf6^GX25^ | *n.d.* | + | 20% | 100% | 100% |

***a***: Tests were carried out similarly as in Table S1.

***b, c****: hs-FLP* and *hs-Cre* stocks used here constitutively express FLPase or Cre recombinase. In *hs-FLP* and *hs-Cre* crosses, constitutively expressed FLPase or Cre will excise the donor DNA, resulting in visible eye color variegation in the cross progeny. The degree of eye color variegation due to the loss of *w+* was compared by estimating the percentage of white area in each eye. 0% white area suggests likely at least one of the two FRT or loxP sites were damaged in the transgenic donor DNA insertion.

***d****: Gal4^2-21^* drives UAS-Rpr expression of the transgenic donor DNA that results in 100% lethality in the cross progeny. Significantly reduced lethality suggests either damaged transgenic donor DNA insertion or severe repression of UAS-Rpr expression due to chromosomal location.

***e****:* These three lines likely have damaged transgenic donor DNA insertion based on their test results with *hs-FLP, hs-Cre* and *Gal4^2-21^*. They also show much higher percentage of *w^[-]^* progeny on 0.25mg/ml G418, presumably due to the fact that reduced G418-resistance in the *w+* progeny allowed better survival of *w^[-]^* siblings.

***f****:* The transgenic donor line used for targeting.

***n.d.***: Not done

**Chr.**: chromosomal location of the transgenic insertion
