# Supplementary material for: W::Neo: A Novel Dual-Selection Marker for High Efficiency Gene Targeting in Drosophila
Source: PLoS One. 2012 Feb 13;7(2):e31997. doi: 10.1371/journal.pone.0031997 (PMC3278458; doi:10.1371/journal.pone.0031997)
Supplement: Table S3 — Optimal culture density for G418 selection in dArf6 targeting. Crosses were first set up in vials, and were then transferred to G418 bottles after one or two days. It appears that between 20 to 40 females per bottle, the yield of w+ candidates per 100 targeting females remains relatively stable. We decided that crosses of 30 targeting females per bottle appears to be a good compromise between achieving the maximum recovery of w+ candidates and minimizing the number of G418 bottles. (DOC) [file pone.0031997.s003.doc]

**Table S3. Optimal culture density for G418 selection in *dArf6* targeting.**

| **Targeting Females** *(per Bottle)* | **# of Bottles** | **Progeny Screened** | **Male Candidates** | | |
| --- | --- | --- | --- | --- | --- |
|  |  |  | *w+* | *w+*% | *w+* Per 100 Mothers |
| 10 | 10 | ~1,200 | 3 | 0.25% | 3 |
| 20 | 10 | ~2,200 | 17 | 0.76% | 8.5 |
| 30 | 10 | ~2,800 | 15 | 0.53% | 5 |
| 40 | 10 | ~5,000 | 25 | 0.50% | 6.3 |
| 50 | 10 | ~3,500 | 16 | 0.46% | 3.8 |
| *Total* | *50* | *~14,700* | *76* | *0.52%* | *5.1* |

Crosses were first set up in vials, and were then transferred to G418 bottles after one or two days. It appears that between 20 to 40 females per bottle, the yield of *w+* candidates per 100 targeting females remains relatively stable. We decided that crosses of 30 targeting females per bottle appears to be a good compromise between achieving the maximum recovery of *w+* candidates and minimizing the number of G418 bottles.
