# Supplementary material for: W::Neo: A Novel Dual-Selection Marker for High Efficiency Gene Targeting in Drosophila
Source: PLoS One. 2012 Feb 13;7(2):e31997. doi: 10.1371/journal.pone.0031997 (PMC3278458; doi:10.1371/journal.pone.0031997)
Supplement: Table S4 — Primers used for the generation and verification of Dscam-N and Dscam-C founder knock-lines. *: Primers for PCR verifications of dArf6 founder lines were in Huang et al [7]. w+: PCR for verifying founder knock-out lines that contain the w+ marker. w[−]: PCR for verifying founder lines that had their w+ marker removed by loxP recombination. n/a: not applicable. (DOC) [file pone.0031997.s004.doc]

**Table S4. Primers used for the generation and verification of *Dscam-N* and *Dscam-C* founder knock-lines.**

| **Gene** | **Primers** | **PCR Product** | |
| --- | --- | --- | --- |
|  |  | **Founder Knock-Out Lines** | **Wild Type (w^1118^)** |
| ***Dscam-N*** |  |  |  |
| 5' homology arm PCR | WK251 CGAGATGCGGCCGCTACTTACTGAAAATGATGCACGTGTC | 5553 bp | 5553 bp |
|  | WK248 CGAGATGCTAGCCACACATAAGCATACTATAAAATGATAAATTC |  |  |
| 3' homology arm PCR | WK255 CGAGATACTAGTTTAAATAAATTTGTAAACTTCGAGCTATTAG | 3226 bp | 3226 bp |
|  | WK254 CGAGATATGCATAACTCTTTCCAAGAGATTCAGCTTT |  |  |
| Long-range 5' PCR-1 (to verify *w+* knockout lines) | WK711 ATCTGACCATAGGAAGTTTGCATAC | 6142 bp | n/a |
|  | WK122 TAGTACAGAGAGGGAGAGTCACAAAA |  |  |
| Long-range 3' PCR-1 (to verify *w+* knockout lines) | WK709 CATTATTACCATCGTGTTTACTGTTTATTG | 3494 bp | n/a |
|  | WK303 GGGCTACGTATTACCTGTTAATGG |  |  |
| Flanking deletion, dPCR1 | WK287 TAAATGGGCCAGACTTGAGCA | 508 bp | 6117 bp |
|  | WK692 TGAACACATAAGAATAGAAACAAATCAA |  |  |
| Inside deletion, dPCR2 | WK627 TTTTATTCGATTCAAAGTCGTTTCA | n/a | 246 bp |
|  | WK407 GGTTGTTGGGACCTAGTGCTG |  |  |
| ***Dscam-C*** |  |  |  |
| 5' homology arm PCR | WK259 CGAGATGCGGCCGCTTACATGGAAGAAAGCAGTTGGTAC | 5302 bp | 5302 bp |
|  | WK257 CGAGATGGTACCGAAAGCACTCAACACATACATATACAAA |  |  |
| 3' homology arm PCR | WK263 CGAGATACTAGTAATCAACTGCCATAAGATCGGAACT | 3188 bp | 3188 bp |
|  | WK262 CGAGATCTCGAGTTAAAACTTCTTCGTCACTTCTAAACAA |  |  |
| Long-range 5' PCR (to verify *w+* knockout lines) | WK305 ATCCGATGCCAAGGTTGAATG | 5901 bp | n/a |
|  | WK708 TTGTTTGTTTGCTCAGCTTGCTT |  |  |
| Long-range 3' PCR (to verify *w+* knockout lines) | WK710 CCCCCTCAAAAAGCTAATGTAATT | 3396 bp | n/a |
|  | WK306 GCCAAGTTTCCCGACTATCAGA |  |  |
| Flanking deletion, dPCR1 | WK289 CTCTAAGCGGATCACAAACAAATT | 564 bp | 8106 bp |
|  | WK307 GTTCGGATAGGTTAAGTGGGAACT |  |  |
| Inside deletion, dPCR2 | WK328 CGAGATGCGGCCGCATGTCTAATGCATTATATTGTTGGTCC | n/a | 252 bp |
|  | WK433 CGAGATCTCGAGGGTCTCCACTGACCGATGCTACGACGT |  |  |

*: Primers for PCR verifications of *dArf6* founder lines were in *Huang et al* [7].

***w+***: PCR for verifying founder knock-out lines that contain the *w+* marker.

***w^[-]^***: PCR for verifying founder lines that had their *w+* marker removed by loxP recombination.

n/a: not applicable.
